# Yield, Quality and Antioxidant Properties of Indian Mustard (*Brassica juncea* L.) in Response to Foliar Biofortification with Selenium and Iodine

**DOI:** 10.3390/plants7040080

**Published:** 2018-09-27

**Authors:** Nadezhda Golubkina, Helene Kekina, Gianluca Caruso

**Affiliations:** 1 Agrochemical Research Center, Federal Scientific Center of Vegetable Production, Moscow Region 143072, Russia; 2 Medical Academy of Postgraduate Education, Moscow 123995, Russia; lena.kekina@mail.ru; 3 Department of Agricultural Sciences, University of Naples Federico II, 80055 Portici (Naples), Italy; gcaruso@unina.it

**Keywords:** selenium, iodine, *Brassica juncea*, foliar biofortification, crop season, element composition, biochemical characteristics

## Abstract

One of the possible ways to challenge selenium (Se) and iodine (I) deficiency in human beings is the joint biofortification of plants with these elements. Though the relationship between Se and I is highly pronounced in mammals, little is known about their interactions in plants where Se and I are considered not to be essential. Peculiarities of Se and I assimilation by a natural Se accumulator, such as *Brassica juncea* L., cultivar Volnushka, were assessed upon joint and separate plant foliar supply with sodium selenate (50 mg Se L^−1^) and potassium iodide (100 mg I L^−1^), in two crop seasons (spring, summer). Conversely to the individual application of Se and I, their joint supply did not stimulate plant growth. Separate use of sodium selenate enhanced I accumulation by 2.64 times, while biofortification with I increased the Se content in plant leaves by 4.3 times; this phenomenon was also associated with significant increase of total soluble solids and ascorbic acid content in leaves. The joint supply of Se and I did not affect the mentioned parameters. Both joint and separate application of Se and I led to synergism between these elements in: inhibiting nitrate accumulation; stimulating flavonoids biosynthesis (2–2.3 times compared to control plants) as well as Al and B accumulation; decreasing Cd and Sr concentrations. Plant biofortification with I increased the content of Mn and decreased K and Li. The consumption of 100 g *Brassica juncea* leaves provided 100% of the adequate human requirement of Se and 15.5% of I.

## 1. Introduction

One of the possible ways to challenge the widespread selenium (Se) and iodine (I) deficiency affecting world population is the joint biofortification of plants with these elements. The essential effects and reciprocal relationships of Se and I in human organism has already been reported [1], whereas Se and I are considered only beneficial elements to plants.

So far the practice of large-scale plant fortification has been carried out only for selenium. Indeed, the use of NPK-fertilizers enriched with sodium selenate in Finland since 1980 contributed to significant decrease of mortality level caused by cancer and cardiovascular diseases, in the absence of environmental pollution due to this element [2]. The latter phenomenon is connected to a large extent with: redox proсesses in soil; formation of insoluble complexes of Se with aluminum as well as iron oxides and hydroxides; microbial reduction of Se to the non-active elemental form and formation of volatile methylated derivatives.

The consumption of carrot, tomato, and lettuce fortified with iodine was proved to be effective in optimizing human iodine status [3]. Similarly to Se, high I lability is connected with both microbiological synthesis of volatile methylated forms and participation in redox reactions. Such processes prevent, to a large extent, the accumulation of toxic concentrations of I in soil, while the ability to form complexes with organic and inorganic matter significantly decreases I bioavailability to plants [4].

Investigations on separate plant fortification with Se or I showed that plant growth is promoted within narrow concentration ranges [4,5]. Conversely to I, Se is supposed to be just essential to plants—hyperaccumulators of this element, able to concentrate up to 4 g Se per kg dry weight without evident symptoms of Se toxicity [6,7].

Some similarities of Se and I biological functions among humans, animals, and plants suggest the existence of close interactions between these elements even in plants. In this respect, Se shows strong antioxidant and immune-modulating properties both in mammals and plants, by protecting the organism against different forms of oxidant stress [8,9]. Moreover, Se reportedly increases plant tolerance to drought, salinity, UV-rays, viral diseases and herbivore, both through antioxidant defense enhancement and acting as an antioxidant directly or indirectly by encouraging the activities of antioxidant enzymes [4,5,10]; both Se and I display strong protection against heavy metals in biological systems [11,12]. Under high Se and I absorptions, all living beings including mammals and plants synthesize specific volatile methylated derivatives [8,13].

The mentioned similarities of Se and I biological functions raise both the need of optimizing the joint I-Se status in humans and of investigating the relationships between these trace elements in plants.

The scant information and, in some available reports, the inconsistent results relevant to plants makes it difficult to gain information about the combined enrichment with Se and I.

A limiting factor for the development of agro-technical methods of Se and I application is the poor recognition of their interaction relevant to plant growth and metabolism. The complexity of this topic includes the selection of the most effective forms of these elements (selenate, selenite, iodate, iodides), the methods of application (hydroponics, soil and foliar application, seeds soaking), the determination of optimal Se and I doses.

The common result of such biofortification is the higher accumulation of Se compared to I, regardless of Se and I chemical form used, which has been reported for soil application of these elements on carrot [14], pumpkin and buckwheat seeds soaking [15], and foliar biofortification of pumpkin and buckwheat sprouts [16].

Among different chemical forms of these elements, Se^+6^ and I^−1^ showed better results on carrot compared to selenate and iodate in soil application [14].

No relationship between Se and I accumulation was recorded in hydroponically grown spinach (Se^+6^, IO_3_^−1^) [17] and in carrot under soil Se and I supply [14]. Conversely, joint fortification of lettuce with IO_3_^−1^ and Se^+6^ in hydroponics showed the stimulating effect of I on Se accumulation [18].

Investigation of interspecies efficiency of plant Se and I fortification led to low differences in iodate and selenate accumulation in winter wheat, maize, soybean, potato, canola, and cabbage in field conditions [19].

The comparison between hydroponics and foliar application of Se^+6^ and IO_3_^−1^ on lettuce showed a higher effectiveness of the latter method [18].

Studies of photosynthetic activity of buckwheat and pumpkin sprouts revealed no effect of the joint Se and I application on seeds soaking [15], but significant changes in flavonoid composition [16].

Notably, though the wide diversity of approaches relevant to Se and I joint enrichment [14,15,16,17,18,19], all the previous investigations have been carried out on non Se-accumulator plants having extremely low tolerance to high concentration of this element.

The aim of the present work was evaluating the responses to the joint biofortification with Se and I of Indian mustard (*Brassica juncea* L.), this species being a well-known Se-accumulator [20]. Other properties of *B. juncea* L., such as fast growth, high intensity of biochemical processes and significant level of biologically-active compounds, proving its medicinal herb properties [21], gave additional strength to the choice of this species for the present investigation.

## 2. Materials and Methods

### 2.1. Plant Material and Experimental Protocol

In 2015 and 2016, a research was conducted on Indian mustard (*Brassica juncea* L.) cv. “Volnushka” at the experimental fields of the Federal Scientific Center of Vegetable Production, Moscow region, Russia (55°39′23″ N, 37°12′43″ E). The trial was carried out in May–June and August–September on a clay-loam soil, with рН 6.8, 2.1% organic matter, 108 mg·kg^−1^ N, 450 mg·kg^−1^ P_2_O_5_, 357 mg·kg^−1^ K_2_O, exchangeable bases sum as much as 95.2%; the average air temperature values, recorded at plant level, were: 13.5 °C in May, 17.1 °C in June, 18.0 °C in August, and 12.2 °C in September.

The experimental protocol, identically repeated in 2015 and in 2016, was based on the factorial combination between two crop seasons (spring, summer) and four foliar biofortification treatments: sodium selenate (50 mg L^−1^ 0.26 mM solution), potassium iodide (100 mg L^−1^ 0.6 mM solution); combined application of sodium selenate (Na_2_SeO_4_) and potassium iodide (KI) at the same concentrations mentioned above; control, with no Se and I application. Foliar application of Se and I is definitely preferable for plant biofortification due to decreasing effect of soil influence, higher absorption rates as a result of large leaf surface, and low ability of I translocation [22], These facts suggest the prospects of leafy vegetables fortification using foliar application of minerals.

In each crop cycle, a randomized complete blocks design with three replicates was used for the treatments distribution in the field, with each treatment covering a 15 m^2^ (5 × 3 m) surface area.

Sowings were performed on 5 May and 2 August along rows spaced 15 cm apart and, after seed germination, the plants were singled out leaving 50 seedlings along each row. The treatments with Se and I solutions were performed over three consecutive days at the phase of eight leaves in June and August. Fourteen days later (16 June and 7 September) the plants were harvested in each plot and the following parameters were assessed: biomass of total plants, leaves and roots; height on fifty plants samples. At the same time, plant samples of each treatment were collected and transferred to the laboratory for analyses, where they were washed with distilled water to remove dust and dried with filter paper.

### 2.2. Antioxidants

#### 2.2.1. Photosynthetic Pigments

Half a g of fresh leaf sample was homogenized in porcelain mortar with 10 mL of ethanol. Homogenized sample mixture was quantitatively transferred to volumetric flask bringing the volume to 25 mL and the mixture was filtered through filter paper. The resulting solution was analyzed for chlorophyll-a, chlorophyll-b, and carotene determination through spectrophotometer UNICO (Unites products & instruments, Prinston, NJ, USA). Calculation of chlorophyll and carotene concentrations was performed using appropriate equations [23]:

Ch-a = 13.36A_664_ − 5.19A_649_; Ch-b = 27.43A_649_ − 8.12A_664_; C c = (1000A_470_ − 2.13Ch-a − 97.632C-b)/209

where A = absorbance, Ch-a = chlorophyll a, Ch-b = chlorophyll b, C c = carotene.

The chlorophyll and carotene contents were expressed in mg·100 g^−1^ fresh weight.

The experiments were carried out in three independent replications.

#### 2.2.2. Ascorbic Acid

It was determined by visual titration of plant extracts in 6% trichloracetic acid with Tillman’s reagent [24]. Three grams of fresh *B. juncea* leaves were homogenized in porcelain mortar with 5 mL of 6% trichloracetic acid and quantitatively transferred to measuring cylinder. The volume was brought to 60 mL using trichloracetic acid, and the mixture was filtered through filter paper 15 min later. The concentration of ascorbic acid was determined from the amount of Tillman’s reagent that went into titration of the sample.

#### 2.2.3. Flavonoids

Total flavonoids content was determined by spectrophotometric method, based on flavonoid-aluminum chloride (AlCl_3_) complexation [25]. The standard of quercetin dehydrate (≥98% HPLC) was purchased from Sigma Co. (St. Louis, MO, USA). Ten mL of methanol were added to 1 g of dried and homogenized samples and the latter were left at room temperature for 2 h. The resulting mixture was filtered through pleated filter. Then, 0.2 mL of the extract was diluted with 1.8 mL of methanol and 0.1 mL of 2% AlCl_3_, 0.5 mL of 1 M sodium acetate solution and 1 mL of distilled water were added. The mixture was incubated for 30 min at room temperature and absorption at 415 nm was measured. Total flavonoid content was determined by standard curve built using five different concentrations of quercetin-AlCl_3_ complex.

### 2.3. Nitrates

They were assessed using ion selective electrode by ionomer Expert-001 (Econix, Moscow, Russia). Five grams of fresh *B. juncea* leaves were homogenized with 50 mL of distilled water. Forty-five milliliters of the resulting extract were mixed with 5 mL of 0.5 M potassium sulfate background solution (necessary for regulating the ionic strength) and analyzed through the ionomer for nitrate determination.

### 2.4. Total Soluble Solids (TSS)

The determination of total soluble solids was carried out on water extracts using portable conductometer TDS-3A (Shenzhen Handsome Technology Co, Shenzhen, China).

### 2.5. Element Composition

Al, As, B, Ca, Cd, Co, Cr, Cu, Fe, Hg, I, K, Li, Mg, Mn, Na, Ni, P, Pb, Se, Si, Sn, Sr, V, and Zn contents in *B. juncea* leaves samples were assessed using ICP-MS on quadruple mass-spectrometer Nexion 300D (Perkin Elmer Inc., Shelton, CT, USA), equipped with the seven-port FAST valve and ESI SC DX4 autosampler (Elemental Scientific Inc., Omaha, NE, USA) at the Biotic Medicine Center (Moscow, Russia). Rhodium 103 Rh was used as an internal standard to eliminate instability during measurements. Quantitation was performed using external standard (Merck IV, multi-element standard solution), potassium iodide for iodine calibration; Perkin-Elmer standard solutions for P, Si, and V, and all the standard curves were obtained at five different concentrations. For quality control purposes, internal controls and reference materials were tested together with the samples on a daily basis. Microwave digestion of samples was performed according to standard method [26] with sub-boiled HNO_3_ (Fluka #02650 Sigma-Aldrich, Co) in the Berghof SW-4 DAP-40 microwave system (Berghof Products + Instruments GmbH, Eningen, Germany), diluted 1:150 with distilled deionized water. Trace levels of Hg and Sn in the samples were not taken into account and, accordingly, they were excluded from the Tables.

The instrument conditions and acquisition parameters were: plasma power and argon flow, 1500 and 18 L·min^−1^ respectively; aux argon flow, 1.6 L·min^−1^; nebulizer argon flow, 0.98 L·min^−1^; sample introduction system, ESI ST PFA concentric nebulizer and ESI PFA cyclonic spray chamber (Elemental Scientific Inc., Omaha, NE, USA); sampler and slimmer cone material, platinum; injector, ESI Quartz 2.0 mm I.D.; sample flow, 637 μL·min^−1^; internal standard flow, 84 μL·min^−1^; dwell time and acquisition mode, 10–100 ms and peak hopping for all analytes; sweeps per reading, 1; reading per replicate, 10; replicate number, 3; DRC mode, 0.55 mL·min^−1^ ammonia (294993-Aldrich Sigma-Aldrich, Co., St. Louis, MO, USA) for Ca, K, Na, Fe, Cr, V, optimized individually for RPa and RPq; STD mode, for the rest of analytes at RPa = 0 and RPq = 0.25.

### 2.6. Statistical Analysis

Data were processed by analysis of variance and mean separations were performed through the Duncan multiple range test, with reference to 0.05 probability level, using SPSS software version 21, IBM, Armonk, NY, USA. Data expressed as percentage were subjected to angular transformation before processing.

## 3. Results and Discussion

Both the year and the season of research had no significant effects on the variables examined, both in terms of main effects and of interactions with the other experimental factor, except for aerial biomass yield and nitrate concentration. Indeed, the values of the two latter variables have been described referring to both the crop seasons, whereas all the other variables are reported as means of the two seasons of research.

### 3.1. Plant Growth and Yield

The present results (Table 1) show that separate application of Se and I increases biomass of both plant aerial parts and roots. In this respect, the growth stimulating effect of Se is connected with the improvement of water supply, increase in chlorophyll biosynthesis as well as S and N assimilation [5,10]; I promotes growth of plants via improvement of N accumulation [4]; both the microelements provide strong plant antioxidant defense.

Furthermore, as it can be seen in Table 1 the joint supply of Se and I did not affect the aerial biomass yield, but increased the ratio of stems to total plant weight. As for the comparison between the two crop cycles, the aerial biomass yield recorded in spring was 14.2% higher than that obtained in summer. Notably, higher stem length was recorded in plants fortified with Se and enhanced dry matter content in stems fortified with I.

Growth stimulating effect of Se on *B. juncea* development was first reported in 1998 [20]; thereafter no attempts have been made to fortify Indian mustard with I.

In the present research, Se and I growth-stimulating effect was recorded only when these trace elements were supplied to plants separately, conversely to their joint application which was ineffective on yield. Consistently with our findings, the lack of plant growth-stimulating effect of combined Se and I application was reported in different crops: lettuce [18], carrot [14], spinach [17]. From the chemical point of view, the lack of growth stimulation effect upon the combined application of Se and I may be connected at least partially with a reaction between selenite (the main Se metabolic derivative in plants) and iodide, with the formation of non-active Se and highly toxic I_2_.

### 3.2. Antioxidants

Se and I in moderate doses display a significant increase of antioxidant activity, which in turn enhances the role of antioxidant enzymes acting as synergists to other antioxidant compounds, such as ascorbic acid, polyphenols, positively affecting photosynthesis [4,5,10]. In general, the effect of Se and I on plant antioxidants accumulation reportedly depends on chemical form of these elements, their concentration and application pattern [22,27].

#### 3.2.1. Photosynthetic Pigments

The increased chlorophyll concentration suggests a higher photosynthetic potential, which in turn is a demonstration of improved plant overall performance. Biofortification of *B. juncea* with Se enhanced chlorophyll b accumulation in leaves, but did not affect chlorophyll a and carotene concentrations. No effect of I on chlorophyll biosynthesis in Indian mustard was recorded instead (Table 2). These results are in accordance with the literature reports about Se involvement in chlorophyll biosynthesis [5,10], the ambiguous effect of foliar enrichment of salad with iodide on the chlorophyll accumulation [28] and lack of effect in other investigations on lettuce seedlings [29].

In our research, the combined application of Se and I reduced the positive influence of Se on chlorophyll biosynthesis and negatively affected carotene concentration in Indian mustard leaves. The decrease of chlorophyll a content caused by the combined application of Se and I in *B. juncea* is in accordance with the data obtained on buckwheat sprouts treated with Se and I [15]. Overall, the results suggest the existence of a weak inhibition effect of the joint Se-I biofortification on chlorophyll a and carotene biosynthesis in Indian mustard.

#### 3.2.2. Ascorbic Acid

The increase of plant antioxidant status as a result of Se or I biofortification has been previously reported [4,5]. Under the conditions of the present investigation, Se and I stimulated the ascorbic acid biosynthesis more intensively in stems than in leaves and the values recorded did not differ from each other. Conversely, the combined plant biofortification with Se and I did not affect the ascorbic acid concentration both in leaves and stems.

#### 3.2.3. Flavonoids

Se reportedly affects the metabolism of nitrogen, protein and amino acid biosynthesis and, in particular, the amino acid phenylalanine is a precursor of phenolics including flavonoids [27]. Indeed, the increase of flavonoids content as a result of Se fortification was detected in tomato [30] and lettuce [31]. Investigations on buckwheat sprouts biofortified with Se and I showed the influence of the chemical form of these elements on grain rutin accumulation [16]. Se at low doses was shown to stimulate the phenylpropanoid metabolic pathway and increase phenol accumulation for overcoming stress conditions [30].

A remarkable effect of I and Se fortification to *B. juncea* is the twofold increase in the content of flavonoids in plant leaves. Similar concentrations of flavonoids in all fortified plants indirectly suggest the independent nature of these elements effect on flavonoid biosynthesis.

### 3.3. Nitrate Accumulation

**Figure 1:**
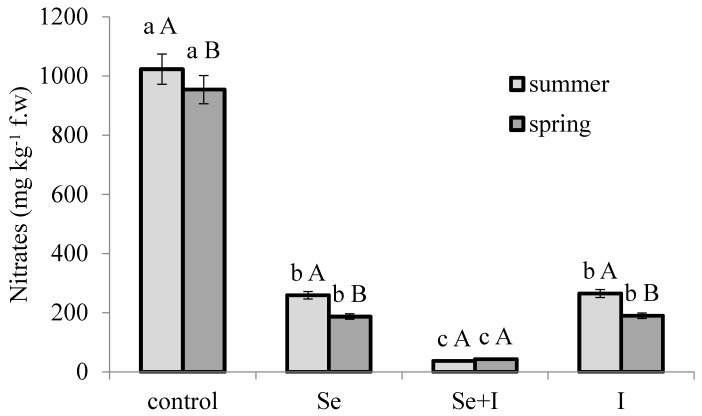
Interaction between crop season and plant biofortification on nitrate accumulation in *Brassica juncea* leaves. Values followed by different letters are significantly different according to the Duncan test at *p* ≤ 0.05: lowercase letters refer to the comparison between biofortification treatments within each crop season; capital letters between crop seasons within each biofortification treatment.

The most impacting phenomenon of *B. juncea* biofortification with Se and I was their strong effect on nitrate accumulation, the latter being 4 times lower under Se and I separate fortifications compared to control plants, but up to 27 times lower upon combined application of the two elements (Figure 1); notably, unlike the other treatments, the application of Se and I resulted in higher nitrate concentration in summer than in spring, which is consistent with the previous reports relevant to the light intensity direct effect on nitrate reductase activity [32]. The concentration of nitrates in plants seems to reflect the imbalance between absorption and assimilation rate. Interestingly, Se is known to stimulate NO_3_^−^ assimilation by enhancing the activity of several enzymes, such as nitrate and nitrite reductases, glutamine synthetase and glutamate synthase [5]. The results provide direct evidence that exogenous Se shows positive function on decreasing NO_3_^−^ accumulation via regulating the transport and enhancing activities of nitrogen metabolism enzyme. Selenium is strictly connected with nitrogen metabolism, by stimulating the amino acid biosynthesis and increasing the nitrate reductase activity [33]. In this respect, the selenate form supplied to potato [34], sunflower [35] and wheat [36] resulted in the increase of nitrate reductase activity and, accordingly, to a decrease of plant nitrate content. However, the effect of Se fortification on nitrate concentration may greatly vary depending on plant hormonal status [37].

Theoretically, in plant nutrient uptake I may compete with other anions such as nitrates, thus reducing the latter accumulation [4]. Nevertheless, controversial results on the relationship between I and nitrates have been reported. Indeed, KI application to plants did not change the nitrate level in radish leaves and roots, though resulting in the increase of free amino acid content [38]. Smolen and Sady [39] reported a strong increase of nitrate accumulation in spinach leaves due to I fortification, whereas the present research showed the opposite effect.

The similarity of Se and I chemical properties suggests that these controversial results may also be connected with plant hormonal changes. In any case, the unexpected strong decrease of nitrate content as a result of joint Se-I supply on *B. juncea* shows the highly synergistic biological effect between Se and I.

### 3.4. Total Soluble Solids (TSS)

**Figure 2:**
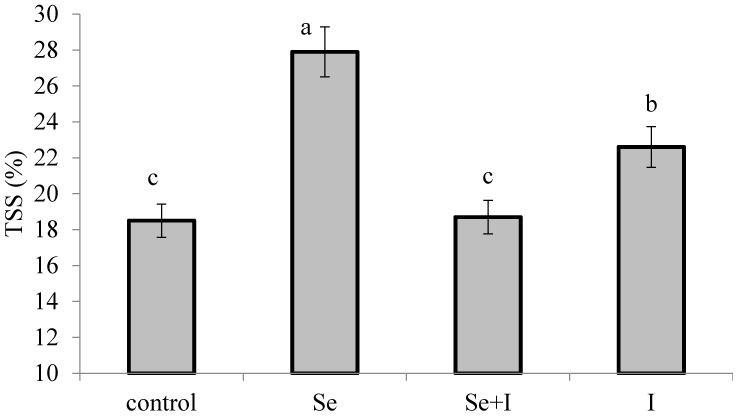
Effect of plant biofortification on TSS content of *B. juncea* leaves. Values followed by different letters are significantly different according to the Duncan test at *p* ≤ 0.05.

Water soluble compounds play a significant role in plant growth and development. Their content reportedly increases as a result of germination [8], suggesting the activation of strong hydrolysis and the consequent synthesis of biologically active compounds. Sugars are the main components of total soluble solids (TSS), playing an important role in carbohydrate metabolism, photosynthesis and production. Leafy vegetable crop plants not producing seeds or other parts that typically accumulate sugars, maintain these sugars in their stems, leaves and roots or employ them for growing up. The highest value of TSS was recorded in *B. juncea* leaves fortified with sodium selenate, with the level decreasing in the plants treated with potassium iodide and with no significant differences between control plants and plants of combined Se-I application (Figure 2). The results presented in Figure 2 are consistent with the growth-stimulating effect of Se and I application.

### 3.5. Biofortification Levels

The scant literature reports on the effect of Se and I on plant elemental composition mainly focused on the effect of Se:I concentration ratio in plant biofortification; in this respect, Smolen et al. [14] found that the most suitable Se:I concentration ratio is 5. In the present research, the concentration of potassium iodide was twofold higher than that of sodium selenate and, in such conditions, the effect of separate Se biofortification was eight times higher than that of I (Table 3). This result may be connected with the high Se accumulation ability of *B. juncea* [5].

The interrelationship between Se and I under separate or joint application to plants is very interesting as showed in Table 3. The related data suggest that, compared to control, the plants fortified with Se had a 2.64-time increase of I content and those enriched with I attained 4.3 times higher concentration of Se. The accumulation of Se in Indian mustard is reportedly increased by the overexpression of genes encoding sulphate transporter *ShST1*, ATP-sulphurylase *AtATPS1* and SeCys methyltransferase *AbSMT* [40]. In this respect, Se-Cys methyltransferase was shown to be overexpressed in lettuce fortified with joint I and Se application [41].

Investigations concerning the I metabolism in plants revealed that I assimilation rate is regulated by chloride transporters, halide methyltransferase (HTM), and halide-thiol transferase (НТМТ), where methyltransferase activity depends on S-adenosyl methionine [4]. However, lack of data relevant to I effect on Se-assimilation enzymes expression and to Se effect on I methyltransferases expression only allows to suppose the existence of similarity between methyltrasferases, which may be partially connected with the similarity between Se and S actions.

On the other hand, the joint application of Se and I showed no synergism between the two elements. A so intensive mutual effect between Se and I may be connected both with the ability of Indian mustard to accumulate high concentrations of Se and with high metabolism rate of this species. In hydroponic experiments on lettuce fortification by foliar supply of selenate and iodate, increase of Se concentration was detected only under the joint Se-I application, compared to the separate supply with Se [15]. However, the latter comparisons are rather complex due to the different doses of the two microelements, different goals of investigation and additional supply of Se and I. The comparison of the present results with literature reports suggests that the stimulation of Se and I content increase recorded in *B. juncea* is not detectable in other Se-accumulators plants, such as *Allium* species. Indeed, foliar application of sodium selenate to wild garlic in the Chechen Republic did not affect I accumulation [42]. Conversely, spraying *A. sativum* with sodium selenate solution in Moscow region decreased I accumulation by garlic bulbs [43]. The involvement of phytohormones may be one of the possible explanations of such controversial results. Moreover, foliar biofortification of spinach with sodium selenate did not change I content in leaves of male spinach forms but decreased I concentration in leaves of female forms [44]. Furthermore, the stimulating effect of salicylic acid on lettuce I accumulation was recorded by Smolen et al. [41] upon combined application of Se and I. Anyway, the study of Se-I interaction mechanism in plants needs additional investigations.

Based on the relevant calculations, it can be inferred that 100 g of fresh Indian mustard leaves fortified with I and Se provide about 100% of the adequate daily consumption level of Se and about 15.5% of I.

### 3.6. Element Composition

Iodine has a significant impact on the redox state of biological systems absorbing this element, which explains its interactions with different organic compounds and metal ions (for instance Fe, Cu, Mn, V), thus modifying the oxidation state and bioavailability [4].

Nevertheless, the effect of Se and I on plant elemental status has not been deeply investigated. In this respect, Se metabolism is connected with the metabolism of P, N, and S, and antagonistic relationships have been found between Se, Hg, and Cd [5]. Moreover, research on spinach biofortification with iodine showed that the synergism or antagonism with other elements depends on the chemical form and dose of this element [11]. Indeed, iodide application to the mentioned species at 1 mg·kg^−1^ soil enhanced the concentration of Mg, Na, and Fe, but reduced Cr; the 50% increase of I dose resulted in higher Na, Fe, Zn, and Al, but lower P, S, and Cu content.

#### 3.6.1. Macroelements

The results of the element analysis of dry *B. juncea* leaves (Table 4) showed a significant Ca decrease under joint or separate plant fortification with I and Se, whereas K decrease occurred only in plants enriched with I. The strongest effect of I on element composition was detected for Na, whose concentration increased 2.5 and three times upon joint Se-I fortification and I supply, respectively. These results are consistent with the reports relevant to the human sodium-iodide symporter overexpression in *Arabidopsis thaliana*, which increases plant concentrations of both I and Na [45]. Consistently with our findings, [18] reported the increase of Na content as a result of I application on lettuce. As it can be seen in Table 4, K:Na ratio significantly decreased as a result of Se and I supply, which is consistent with previous reports regarding Na effect on K:Na ratio in *B. juncea* [45,46]. Finally, no effect of I on Mg and P accumulation has been recorded in Indian mustard.

#### 3.6.2. Trace Elements

As reported in Table 5, the Se-I fortification effect on the *B. juncea* element composition was significant. Indeed, the foliar enrichment of Indian mustard with Se and I resulted in the increase by 1.25–1.58 times of B and by 1.23–1.38 times of Со content, both under joint and separate biofortification with the two elements (Table 5). The phenomenon may be of high practical significance as *B. juncea* is known to be very sensitive to micronutrient deficiency, particularly referred to boron [46,47]. Moreover, the joint application of Se and I resulted in 1.5 time increase of Li and B in Indian mustard leaves, whereas Mn accumulation was only affected by I supply. Notably, these results are consistent with the reports of Smolen et al. [48], who have found that I fortification of hydroponically-grown tomato increases the concentration of Na, B and Mn in leaves and fruits. In this respect, the positive effect of I on Mn accumulation may be connected with the fact that greater amount of iodine in plant tissues encourages the activity of the enzymatic systems dissipating this element, such as Mn-dependent oxidases [4].

The heterogeneity of the results concerning the influence of Se and I on the plant elemental composition may be partially connected with the hormonal regulation, as previously reported for male and female forms of spinach [44].

#### 3.6.3. Heavy Metals

As it can be observed in Table 6, separate and joint application of Se and I on *B. juncea* decreased the accumulation of Cd and Sr, but increased Al concentration. The latter result is consistent with the previous reports concerning the sodium selenate effect on spinach element composition [44], and potassium iodide effect on lettuce [18]. Moreover, statistically significant increase of V in Indian mustard leaves was recorded only in plants separately treated with Se and I. The data reported in Table 6 suggest the practical significance of the Se and I biofortification of *B. juncea*, due to this species ability to decrease the ecological risks connected with the heavy metals and radio nuclides environmental contamination. Notably, the short crop cycle of *B. juncea* and its high ability to accumulate heavy metals (Pb, Cd, Cr) make this species very interesting for soil phytoremediation [47,49].

## 4. Conclusions

The overall results of the present research allowed to record important effects of *B. juncea* fortification with I and Se: enhanced accumulation of Se and I under separate foliar application of these elements; significant synergistic effect of Se and I on nitrate reductase activity; stimulating effect of both Se and I on flavonoid content in plant leaves; significant effect on the content of Na, Al, Co, V, B, Mn, Sr, Ca, and K. As plant growth was not inhibited by the joint Se-I application, the production of Indian mustard using high concentrations of I and Se represents an interesting target.

## Figures and Tables

**Table 1:** Effect of plant biofortification on biometrical parameters and dry matter content of *B. juncea* in spring and summer crop cycles.

Parameter		Control	Se	Se + I	I
**Spring**					
Aerial biomass yield (g·plant^−1^)		33.4 ± 3.8 ^b^	51.5 ± 4.7 ^a^	30.6 ± 5.0 ^b^	45.8 ± 5.6 ^a^
Ratio of leaves to the aerial biomass (%)		55.8	57.5	48.2	51.1
Root biomass (g·plant^−1^)		1.48 ± 0.47 ^ab^	2.29 ± 0.48 ^a^	1.31 ± 0.40 ^b^	2.25 ± 0.37 ^a^
Plant height (cm)		54.9 ± 8.4 ^b^	61.3 ± 10.0 ^ab^	71.9 ± 7.2 ^a^	60.9 ± 7.1 ^ab^
Dry matter (%)	Leaves	8.2 ± 0.3 ^b^	9.2 ± 0.2 ^a^	8.3 ± 0.1 ^b^	9.1 ± 0.1 ^a^
	Stems	4.5 ± 0.1 ^c^	4.8 ± 0.1 ^b^	5.0 ± 0.1 ^ab^	5.2 ± 0.1 ^a^
**Summer**					
Aerial biomass yield (g·plant^−1^)		30.5 ± 3.5 ^b^	45.0 ± 4.7 ^a^	25.5 ± 4.0 ^b^	40.2 ± 4.4 ^ab^
Ratio of leaves to the aerial biomass (%)		56.1	57.0	49.2	50.1
Root biomass (g·plant^−1^)		1.40 ± 0.41 ^bc^	2.40 ± 0.54 ^a^	1.20 ± 0.40 ^c^	2.20 ± 0.55 ^ab^
Plant height (cm)		55.0 ± 8.2 ^b^	60.4 ± 11.0 ^ab^	72.2 ± 8.1 ^a^	57.5 ± 6.7 ^ab^
Dry matter (%)	Leaves	9.1 ± 0.1 ^a^	8.2 ± 0.1 ^b^	9.2 [g23]± 0.2 ^a^	9.1 ± 0.1 ^a^
	Stems	4.8 ± 0.1 ^b^	5.1 ± 0.1 ^a^	5.1 ± 0.1 ^a^	5.2 ± 0.1 ^a^

Mean ± standard deviation of each variable is reported in correspondence with each experimental treatment. Along each line, values followed by different letters are significantly different according to the Duncan test at *p* ≤ 0.05.

**Table 2:** Effect of plant biofortification on the antioxidant profile of *B. juncea* leaves.

Parameter	Control	Se	Se + I	I
Chlorophyll a (mg·100 g^−1^ f. w.)	6.97 ± 0.6 ^a^	7.30 ± 0.7 ^a^	5.73 ± 0.5 ^b^	8.22 ± 0.7 ^a^
Chlorophyll b (mg·100 g^−1^ f. w.)	2.92 ± 0.3 ^b^	3.98 ± 0.4 ^a^	2.65 ± 0.3 ^b^	2.92 ± 0.3 ^b^
Carotene (mg·100 g^−1^ f. w.)	1.24 ± 0.1 ^ab^	1.38 ± 0.1 ^a^	1.03 ± 0.1 ^c^	1.13 ± 0.1 ^bc^
Leaf ascorbic acid (mg·100 g^−1^ f. w.)	88 ± 2 ^b^	97 ± 2 ^a^	92 ± 3 ^ab^	93 ± 3 ^ab^
Stem ascorbic acid (mg·100 g^−1^ f. w.)	26 ± 1 ^b^	32 ± 1 ^a^	24 ± 1 ^b^	31 ± 1 ^a^
Flavonoids (mg·100 g^−1^ f. w.)	31.2 ± 2.5 ^b^	69.7 ± 7.3 ^a^	63.6 ± 7.1 ^a^	70.9 ± 5.1 ^a^

Mean ± standard deviation of each variable is reported in correspondence with each experimental treatment. Along each line, means followed by different letters are significantly different according to the Duncan test at *p* ≤ 0.05.

**Table 3:** Accumulation of Se and I in *B. juncea* plants.

Parameter	Control	Se	Se + I	I
Se content (µg·kg^−1^ d.w.)	60 ± 6 ^c^	8922 ± 870 ^a^	8577 ± 624 ^a^	258 ± 21 ^b^
Se biofortification level	-	149	143	4.3
I content (µg·kg^−1^ d.w.)	183 ± 18 ^c^	483 ± 48 ^b^	2790 ± 279 ^a^	3412 ± 341 ^a^
I biofortification level	-	2.64	15.2	18.6

Mean ± standard deviation of each variable is reported in correspondence with each experimental treatment. Along each line, values followed by different letters are significantly different according to the Duncan test at *p* ≤ 0.05.

**Table 4:** Effect of plant biofortification on macroelement content of *B. juncea* leaves (g Kg^−1^ d.w.).

Element	Control	Se	Se + I	I
Ca	21.0 ± 2.1 ^a^	16.7 ± 1.7 ^b^	14.5 ± 1.5 ^b^	17.4 ± 1.7 ^ab^
K	43.3 ± 4.3 ^a^	37.6 ± 3.8 ^ab^	30.5 ± 3.0 ^b^	34.0 ± 3.4 ^b^
Mg	2.82 ± 0.28 ^a^	2.54 ± 0.25 ^a^	2.55 ± 0.25 ^a^	3.04 ± 0.30 ^a^
Na	0.72 ± 0.07 ^b^	0.80 ± 0.08 ^b^	2.21 ± 0.22 ^a^	1.86 ± 0.19 ^a^
P	6.22 ± 0.62 ^a^	6.33 ± 0.63 ^a^	7.35 ± 0.74 ^a^	6.04 ± 0.60 ^a^
K:Na	60	47	14	18

Mean ± standard deviation of each variable is reported in correspondence with each experimental treatment. Along each line, values followed by different letters are significantly different according to the Duncan test at *p* ≤ 0.05.

**Table 5:** Effect of plant biofortification on trace elements content of *B. juncea* leaves (mg Kg^−1^ d.w.).

Element	Control	Se	Se + I	I
B	16.29 ± 1.63 ^c^	20.34 ± 2.03 ^b^	25.70 ± 2.57 ^a^	21.20 ± 2.12 ^ab^
Co	0.13 ± 0.02 ^b^	0.18 ± 0.02 ^a^	0.16 ± 0.02 ^ab^	0.16 ± 0.02 ^ab^
Fe	182 ± 18 ^a^	199 ± 20 ^a^	181 ± 18 ^a^	206 ± 21 ^a^
Li	0.16 ± 0.02 ^bc^	0.18 ± 0.02 ^b^	0.24 ± 0.03 ^a^	0.12 ± 0.03 ^c^
Mn	23.96 ± 2.40 ^c^	29.30 ± 2.93 ^bc^	33.25 ± 3.32 ^b^	48.29 ± 4.83 ^a^
Si	170 ± 17 ^a^	149 ± 15 ^a^	167 ± 17 ^a^	140 ± 14 ^a^
Zn	36.0 ± 3.6 ^a^	34.2 ± 3.4 ^a^	39.6 ± 4.0 ^a^	40.2 ± 4.0 ^a^

Mean ± standard deviation of each variable is reported in correspondence with each experimental treatment. Along each line, values followed by different letters are significantly different according to the Duncan test at *p* ≤ 0.05.

**Table 6:** Effect of plant biofortification on heavy metals content of *B. juncea* leaves (mg Kg^−1^ d.w.).

Element	Control	Se	Se + I	I
Al	87 ± 9 ^b^	125 ± 13 ^a^	117 ± 12 ^a^	123 ± 12 ^a^
As	0.10 ± 0.02 ^a^	0.14 ± 0.02 ^a^	0.13 ± 0.02 ^a^	0.13 ± 0.02 ^a^
Cd	0.32 ± 0.04 ^a^	0.15 ± 0.02 ^c^	0.17 ± 0.02 ^bc^	0.20 ± 0.02 ^b^
Cr	0.64 ± 0.08 ^a^	0.70 ± 0.08 ^a^	0.60 ± 0.07 ^a^	0.68 ± 0.08 ^a^
Cu	8.45 ± 0.85 ^a^	7.61 ± 0.76 ^a^	8.55 ± 0.85 ^a^	8.71 ± 0.87 ^a^
Ni	1.69 ± 0.17 ^a^	1.43 ± 0.14 ^a^	1.59 ± 0.16 ^a^	1.58 ± 0.16 ^a^
Pb	0.82 ± 0.10 ^a^	1.03 ± 0.10 ^a^	0.94 ± 0.11 ^a^	0.93 ± 0.11 ^a^
Sr	96.6 ± 9.7 ^a^	64.2 ± 6.4 ^b^	57.0 ± 5.7 ^b^	68.1 ± 6.8 ^b^
V	0.26 ± 0.03 ^c^	0.35 ± 0.04 ^a^	0.31 ± 0.04 ^ac^	0.34 ± 0.04 ^ab^

Mean ± standard deviation of each variable is reported in correspondence with each experimental treatment. Along each line, values followed by different letters are significantly different according to the Duncan test at *p* ≤ 0.05.
